# Correction: Wu et al. Nano-Silicon@Exfoliated Graphite@Pyrolytic Polyaniline Composite of a High-Performance Anode for Lithium Storage. *Materials* 2023, *16*, 1584

**DOI:** 10.3390/ma19143126

**Published:** 2026-07-21

**Authors:** Qian Wu, Yinghong Zhu, Haojie Duan, Lin Zhu, Yuting Zhang, Hongqiang Xu, Ishioma Laurene Egun, Haiyong He

**Affiliations:** 1 College of Chemical Engineering, Zhejiang University of Technology, Hangzhou 310014, China; wuqian20@nimte.ac.cn (Q.W.); yhzhuchem@zjut.edu.cn (Y.Z.); 2 Ningbo Institute of Materials Technology and Engineering, Chinese Academy of Sciences, Ningbo 315201, China; duanhaojie@nimte.ac.cn (H.D.); zhulin@nimte.ac.cn (L.Z.); zhangyuting@nimte.ac.cn (Y.Z.); xuhongqiang@nimte.ac.cn (H.X.); ishioma.egun@nimte.ac.cn (I.L.E.); 3 Department of Chemical and Environmental Engineering, Faculty of Science and Engineering, University of Nottingham Ningbo China, Ningbo 315100, China

## Errors in Figure and Figure Legend

In the original publication [1], there were mistakes in Figures 2 and 3, as well as in the legend for Figure 3. The corrected Figure 2 and Figure 3 and the corrected legend for Figure 3 are shown below:

## Text Corrections

There were some errors in the original publication [1]. Some text needed to be updated in the title, and in Sections 2–4.

The article title “Nano-Silicon@Exfoliated Graphite/Pyrolytic Polyaniline Composite of a High-Performance Cathode for Lithium Storage” should be updated to “Nano-Silicon@Exfoliated Graphite@Pyrolytic Polyaniline Composite of a High-Performance Anode for Lithium Storage”.

In Section 2, paragraph 2, the sentences “The hydrochloric (200 mg, AR: 98%) aqueous solution and aniline monomer (AR: 99.5%) (molar mass 1:20) were dripped into the above suspension. After that, a certain amount of ammonium persulfate (APS, AR: 99.99%) oxidant was dissolved into 40 mL of 1 M HCL solution that was precooled in advance, and then it was added into the abovementioned mixed solution. The molar mass ratio of APS/aniline is 3:2, and it is continuously stirred in an ice-water bath for 24 h until the color of the solution changes to dark green. The dark green product was filtered and washed three times with ethanol, and the obtained filter cake was dried in a vacuum oven at 60 °C for 12 h. The resultant product was denoted as Si@EG@p-PANI after being calcined at 700 °C in an argon atmosphere for 7 h.” should be updated to the following version:

“The hydrochloric acid (200 mg, AR: 36–38%) aqueous solution and aniline monomer (AR: 99.5%) (molar mass ratio 1:20) were dripped into the above suspension. After that, a certain amount of ammonium persulfate (APS, AR: 98%) oxidant was dissolved into 40 mL of 1 M HCl solution that was precooled in advance, and then it was added into the abovementioned mixed solution. The molar mass ratio of APS/aniline is 3:2, and the mixture was continuously stirred in an ice-water bath for 24 h until the color of the solution changed to dark green. The dark green product was filtered and washed three times with ethanol, and the obtained filter cake was dried in a vacuum oven at 60 °C for 12 h. The resultant product was denoted as Si@EG@p-PANI after being calcined at 700 °C in an argon atmosphere for 7 h.”

The title of Section 3, “3. Results”, should be updated to “3. Results and Discussion”.

The title of Section 4, “4. Discussion”, should be updated to “4. Conclusions”.

The authors state that the scientific conclusions are unaffected. This correction was approved by the Academic Editor. The original publication has also been updated.

## Figures and Tables

**Figure 2:**
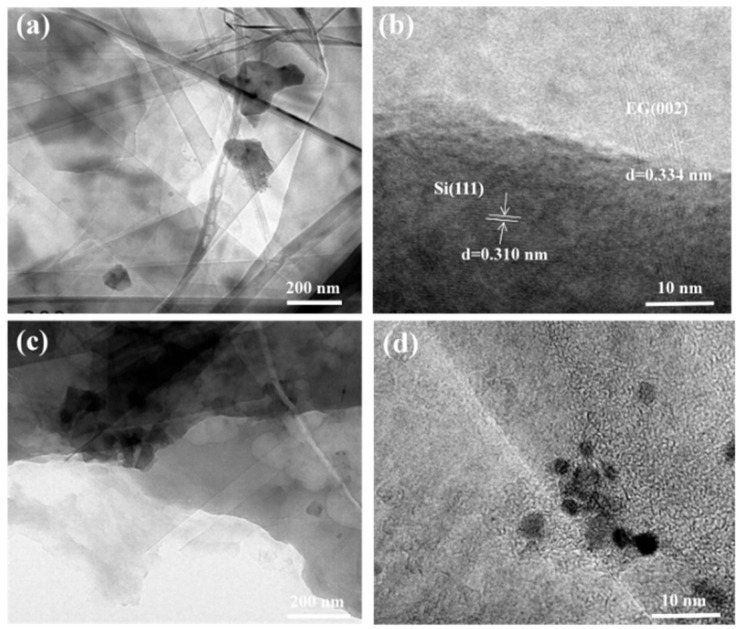
(**a**) TEM and (**b**) HRTEM images of Si@EG; (**c**) TEM and (**d**) HRTEM images of Si@EG@p-PANI composites.

**Figure 3:**
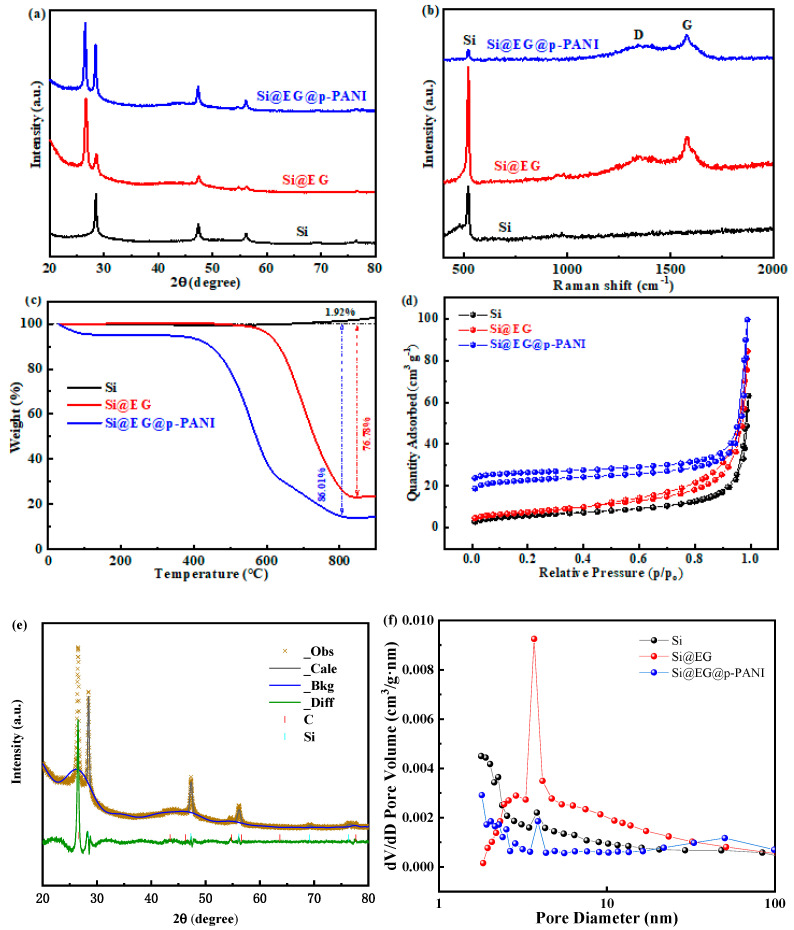
(**a**) XRD patterns, (**b**) Raman spectra, and (**c**) TGA curves of raw Si powder, Si/EG composite, and Si/EG@p-PANI composite in air at a heating rate of 5 °C min^−1^, and (**d**) N_2_ adsorption and desorption isotherm of raw Si powder, Si/EG composite, and Si/EG@p-PANI composite. (**e**) Rietveld refinement of XRD patterns and (**f**) pore size distribution.
